# UHPLC-MS/MS Determination, Pharmacokinetic, and Bioavailability Study of Taxifolin in Rat Plasma after Oral Administration of its Nanodispersion

**DOI:** 10.3390/molecules21040494

**Published:** 2016-04-14

**Authors:** Chun-Juan Yang, Zhi-Bin Wang, Ying-Ying Mi, Ming-Jie Gao, Jin-Nan Lv, Yong-Hai Meng, Bing-You Yang, Hai-Xue Kuang

**Affiliations:** 1 College of Pharmacy, Harbin Medical University, No. 157 Baojian Road, Nangang District, Harbin 150081, Heilongjang, China; chunjuanyang@126.com (C.-J.Y.); gaomingjie8888@163.com (M.-J.G.); 2 Key Laboratory of Chinese Materia Medica (Ministry of Education), Heilongjiang University of Chinese Medicine, Harbin 150040, Heilongjang, China; ccmini731@163.com (Y.-Y.M.); liyufeng5211314@126.com (J.-N.L.); 15845002546@139.com (Y.-H.M.); ybywater@163.com (B.-Y.Y.)

**Keywords:** taxifolin, UHPLC-MS/MS, nanodispersion, ratplasma, pharmacokinetics

## Abstract

A rapid and sensitive LC-MS/MS method based on the Triple Quad system has been developed and validated for the determination and pharmacokinetics of taxifolin and its nanodispersion in rat plasma. Taxifolin plasma samples along with butylparaben (internal standard) were pre-treated by liquid-liquid extraction with ethyl acetate, and then separated on a SB-C_18_ RRHD column (150 mm × 2.1 mm × 1.8 μm) using isocratic elution with a run time of 3.0 min. The mobile phase was acetonitrile–water (90:10, *v/v*) containing 5 mM ammonium acetate at a flow rate of 0.4 mL/min. Quantification of taxifolin was performed by the electrospray ionization tandem mass spectrometry in the multiple reaction monitoring (MRM) mode with negative atmospheric ionization at *m/z* 303.0→285.0 for taxifolin and 193.1→92.0 for I.S., respectively. The calibration curve of taxifolin showed good linearity over a concentration range of 5.0–4280 ng/mL with a correlation coefficient of 0.9995. The limit of quantification (LLOQ) was 5.0 ng/mL. Intra-day, inter-day precision and accuracy (percent relative to standard deviation) were all within 8% at three concentration levels. A total recovery of taxifolin and I.S. was beyond 75%. The present LC-MS/MS method was successfully applied to pharmacokinetic studies of taxifolin after intravenous administration of taxifolin, oral administration of its physical mixture and nanodispersion. The absolute bioavailability of taxifolin was calculated as 0.75% for taxifolin nanodispersion and 0.49% for taxifolin, respectively.

## 1. Introduction

Taxifolin (5,7,3′,4′-tetrahydroxyflavanol, dihydroquercetin) belongs to a member of the flavonoids family. Taxifolin was found from the leaves of *Chamaecyparis obtuse* (Cupressaceae) and also commonly extracted from *Pseudotsuga taxifolia* (Lamb.) Britt., *Larix gmelinii* (Rupr.) Kuzen, and *Larix sibirica* Ledeb. (Pinaceae) [1,2,3]. It was even obtained from fruits, vegetables, beverages, and so on. It elicits a wide range of pharmacological effects of anti-oxidation and anti-radiation [4,5]. Furthermore, it also has anti-inflammation activity, anti-viral activity, anti-tumor activity, and protective postmenopausal osteoporosis activity [6,7,8,9]. Due to its pharmacological diversity, its bioavailability and biological properties have raised a great interest in future studies.

Flavonoids, including taxifolin, are slightly soluble in water and show a slow dissolution rate from solid oral dosage forms, restricting their clinical use. The poor solubility of active pharmaceutical ingredients in water and their low dissolution rate in the aqueous gastro-intestinal fluids often leads to insufficient bioavailability, which becomes one of the most difficult problems in pharmaceutical technology. The dissolution of poor water-soluble drugs that undergo dissolution rate-limited gastrointestinal absorption can generally be improved by many techniques, one of which is the preparation of nanodispersion [10,11,12]. This technology provides the possibility to reduce the drug particle size and polyvinylpyrrolidone (PVP) selected as the carrier, increasing the surface area and, hence, improving the dissolution rates [13,14]. In our previous study, taxifolin nanodispersion was prepared by dissolvent-fusion method with PVP to improve the dissolution of taxifolin [14,15].

There are several papers reported for the determination of taxifolin in plasma samples, and several analytical methods have been developed for the determination of taxifolin in plasma samples in the previous reports. A HPLC-UV method has been reported for the determination and pharmacokinetic study of taxifolin in rabbit plasma with a limit of quantitation (LOQ) of 110 ng/mL. Few HPLC-MS methods with better sensitivities have been reported for quantitation of taxifolin in rat plasma [16], in beagle dog plasma [17], and in rabbit plasma [18]. Taxifolin nanoparticles has been introduced due to the enhancement solubility, antioxidant, and bioavailability [19]. In this paper, we have developed and validated a UPLC-MS/MS method to quantify taxifolin in rat plasma. The test method is sensitive with a LOQ of 5 ng/mL, and the run time is only 3 min for each injection. It is the first time that a fully-validated analytical method has been applied to the pharmacokinetic study of taxifolin after oral administration of taxifolin and its nanodispersion.

## 2. Results and Discussion

### 2.1. LC–MS/MS Analysis

Both positive and negative-ion detection modes were tried to optimize ESI conditions for detecting taxifolin and internal standard. The intensity of signal of negative ionization mode was more stable and stronger than positive ionization mode. Finally, the negative ion mode was employed. In order to establish a MS quantitative method, the first step was to do a full scan of taxifolin and I.S. so as to ascertain their precursor ions and to select product ions for the use in MRM mode. The second step was to optimize MS parameters, such as fragmentor, collision energy and cell accelerator voltage to get the richest relative abundance of precursor and product ions. The LC–MS/MS method has high specificity because specific ion pairs from analytes were monitored. The *m*/*z* 303→285 for taxifolin and *m*/*z* 193→92 for I.S were chosen for MRM transitions. In order to make this analytical method more accurate, we used the standard of taxifolin and I.S. to find the qualifier ion. The *m*/*z* 303→125 for taxifolin and *m*/*z* 193→136 for I.S were chosen as qualifier, respectively. Wang *et al.* chose the SIR mode as the as the detection mode and the concentration of LLOQ was 6 ng/mL [18]. In our study the MRM mode was chosen as the concentration of LLOQ 5 ng/mL. The key step was to select a proper internal standard which could improve the method performance, ultimately butylparaben was chosen as the I.S. which was similar to taxifolin in the aspect of chromatographic behavior, ionization response, and extraction efficiency.

According to the analysis method presented in the literature [20], chromatographic conditions were optimized to improve peak shape, increase sensitivity, and optimize the run time for simultaneous analysis of taxifolin and I.S. Compared with the HPLC column, we chose the UHPLC column as separation system, in which the particle size of stationary phase in UHPLC column is smaller. Therefore, the resolution, sensitivity, and analysis time will be greatly improved. Several mobile phase conditions such as mobile phase systems methanol–water and acetonitrile–water, and different buffers including ammonium acetate (2 and 5 mM), formic acid (0.1%), and acetic acid (0.2%) were tested. Finally, acetonitrile–water (90:10, *v*/*v*) and 5 mM ammonium acetate in water were selected as best solvent composition. Satisfactory separation was achieved in 3 min by isocratic elution using a C_18_ column (SB-C18 RRHD 150 mm × 2.1 mm, 1.8 μm) at a flow rate of 0.4 mL/min. The retention time of taxifolin was 0.9 min in this study. In the literature, however, the retention time of taxifolin was 4.4 min [20].

### 2.2. Method Validation

#### 2.2.1. Specificity and Selectivity

**Figure 1:**
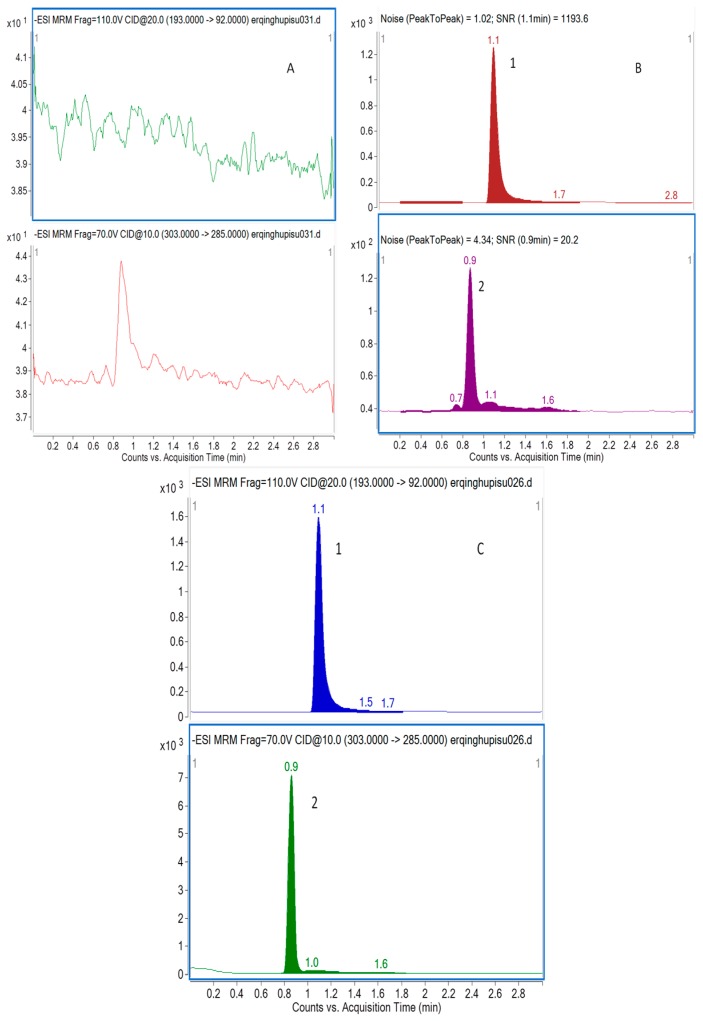
Representative MRM chromatograms of taxifolin (2) and I.S. (1) in rat plasma (**A**) a blank sample (without taxifolin and I.S.); (**B**) LLOQ sample (taxifolin at 5.0 ng/mL and I.S. at 1000 ng/mL); and (**C**) a rat sample taken 0.5 h after administration of 15 mg/kg nanodispersion of taxifolin.

The selectivity of the method towards endogenous plasma matrix was evaluated with plasma from six rats. Two channels were used for recording and the retention times of taxifolin and I.S. were 0.9 min and 1.1 min, respectively. Typical chromatograms were obtained from a blank, and a spiked plasma sample with the analytes and I.S. (LLOQ), and 0.5 h plasma sample after an oral administration of taxifolin, oral administration of physical mixture, and nanodispersion of taxifolin are shown in Figure 1. The peak of analyte and I.S. were detected with excellent resolution, as well as peak shapes, and no interference from the endogenous substances was observed at the retention time of the analytes and I.S. The analytes could be easily differentiated from the rat plasma matrix and quantitatively determined at the LLOQ level.

#### 2.2.2. Linearity, Sensitivity, Matrix Effect, and Extraction Recovery

The calibration curve was conducted by plotting the peak-area ratio (y) of taxifolin to the I.S. *versus* the plasma concentration (x) of taxifolin. The mean linear regression equation and correlation coefficient (r) for the calibration curve was shown as follows: y = 0.01038 x + 0.02114 (r = 0.9995). The lower limit of quantification (LLOQ) of taxifolin was 5 ng/mL (signal- to- noise ≥20). The limit detection (LOD) of taxifolin with a signal-to-noise ratio of 5 was 1.5 ng/mL.

The summary of matrix effect and extraction recovery of the analytes is shown in Table 1. Mean absolute recoveries of taxifolin under the liquid–liquid extraction conditions at three QC levels with six samples were from 78.5% to 83.5% for taxifolin and 85.8% for I.S. The extraction solvent used in the experiment showed good extraction efficiency. The mean recoveries of taxifolin and I.S. were all above 75.0% which indicated that recoveries were consistent, precise, and reproducible at different concentrations. All the variations of the matrix effects ranged from −0.8% to 8.5%. These results confirmed that evaluated method was free from any matrix effect.

#### 2.2.3. Precision, Accuracy, and Stability

In this assay, the intra-day, inter-day precision and accuracy were determined by analyses of QC samples at three concentration levels (LQC, MQC, and HQC) of taxifolin on the same day and three different days. The results are listed in Table 2 which indicated the intra and inter-day precisions (R.S.D) of these analytes ranged from 3.3% to 7.4% and 2.1% to 5.0%, respectively. The accuracy was at range of −2.5% to 3.8% for intra-day and −5.6% to 2.7% for inter-day, respectively. The results above were all within the permissible criteria of ±15%, which suggested that the method was accurate and reproducible for the determination of taxifolin in rat plasma. A summary of the stability of taxifolin under different conditions was shown in Table 3. This indicated that taxifolin was stable in plasma after three freeze-thaw cycles, at room temperature for 4 h. Post-preparative stability of the analytes also showed that no significant degradation occurred when the extracted samples were kept at 4 °C for 12 h. Moreover, the investigated compound was stable for two weeks when kept frozen at −20 °C.

### 2.3. Pharmacokinetics Analysis

**Figure 2:**
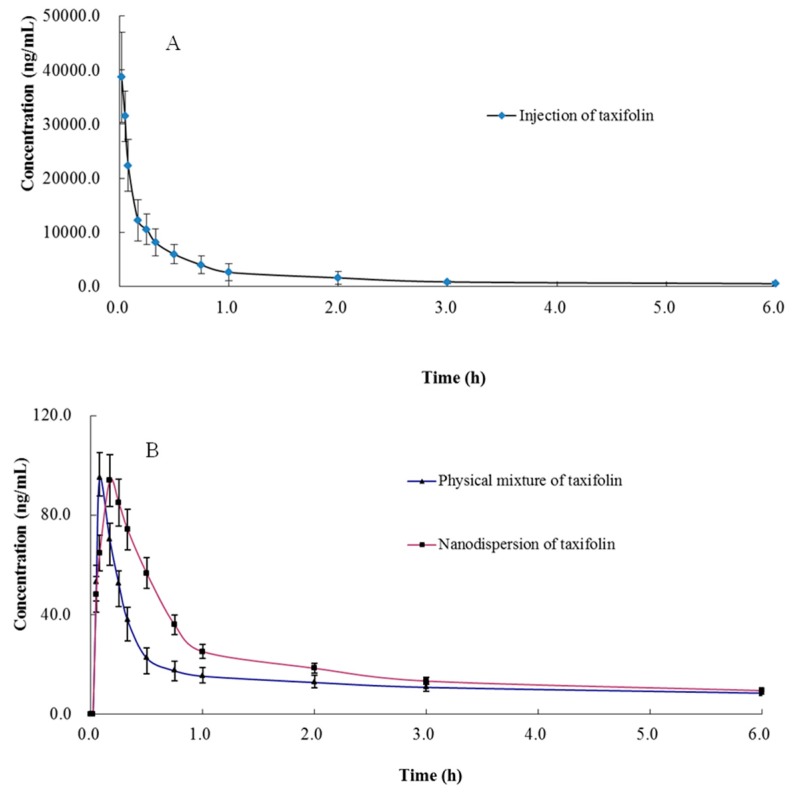
Mean ± SD (*n* = 6) plasma concentration–time curves of taxifolin (**A**) intravenous administration of taxifolin at 15 mg/kg (**B**) oral administration of physical mixture of taxifolin and nanodispersion of taxifolin 15 mg/kg.

The validated method was successfully applied to pharmacokinetic studies of taxifolin in rat plasma after intravenous administration of taxifolin and an oral administration of a physical mixture of taxifolin and its nanodispersion with the same dose of 15 mg/kg. It has been reported that there was an obvious species difference of the bioavailability among rabbit, rat, and dog [14,17,18]. Zu *et al.* developed a method to roughly analyze the bioavailability of taxifolin nanoparticles [19]. However, in our study, not only have we described the pharmacokinetic characteristics of taxifolin in rat plasma after intravenous and oral administration of taxifolin, but also found the differences of bioavailability between physical mixture of taxifolin and its nanodispersion in detail. Compared with the literature (*n* = 3), the number of experimental animals in this study (*n* = 6) is more statistically meaningful. In addition, the whole method verification was carried out in our research, which can provide appropriate analytical methods for others. The summary of main pharmacokinetic parameters of taxifolin is shown in Table 4. The mean plasma concentration-time curves (*n* = 6) of the taxifolin are shown in Figure 2. According to the charts we could see that there were great differences between two routes (intravenous and oral administration) for t_1/2_, which was consistent with the literature [14,17]. The result implied that the bioavailability of taxifolin after oral administration of nanodispersion (0.75%) was much higher than that of oral administration of taxifolin (0.49%), and the AUC_0_-t of nanodispersion of taxifolin (90.89 ± 11.76 ng h/mL) is significantly higher than that of the physical mixture of taxifolin after oral administration (59.11 ± 8.62 ng h/mL) (*p* < 0.001). The higher bioavailability of taxifolin in nanodispersion form would due to the improvement of taxifolin solubility and result in a more efficient absorption.

## 3. Experimental Section

### 3.1. Chemicals and Reagents

**Figure 3:**
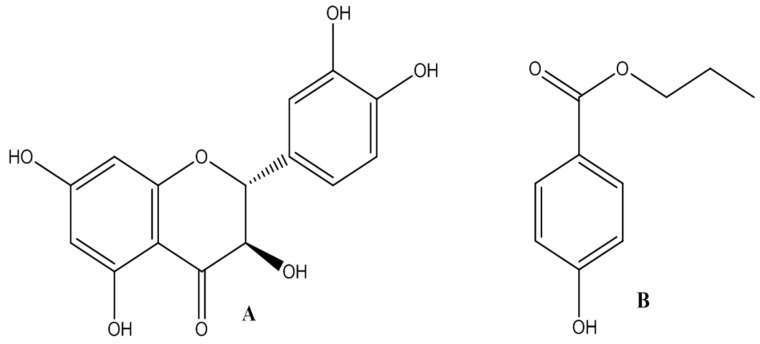
Chemical structures of taxifolin (**A**) and butylparaben (**B**).

Taxifolin (C_15_H_12_O_7_, M_W_ = 304.0, purity, 99.0%) was supplied by Chinese Chemical Laboratory of Heilongjiang University of Chinese Medicine (Harbin, China). The nanodispersion of taxifolin was manufactured strictly according to the literature [14]. Butylparaben (C_11_H_14_O_3_, M_W_ = 194.23, purity, 99.0%) was purchased from Guangfu Fine Chemical Research Institute (Tianjin, China) as the internal standard (I.S.) for UHPLC-MS/MS and pharmacokinetic analysis of taxifolin. The chemical structures of taxifolin and butylparaben are given in Figure 3. Acetonitrile of LC-MS-grade was purchased from Thermo Fisher Scientific (Shanghai, China). Ammonium acetate (purity ≥ 98.0%) of HPLC-grade was purchased from Tianjin Kermel Chemical Reagent (Tianjin, China). High-purity nitrogen (purity, 99.999%) and liquid nitrogen at the same purity grade were purchased from Dawn Gas (Harbin, China). Ultra-pure water used throughout the experiment was prepared from Milli-Q water purification system (Millipore Corp, Bedford, OH, USA). All of the other reagents and solvents were of analytical grade.

### 3.2. Animals

Wistar rats (male, 200 ± 15 g) were supplied by Center for Drug Safety Evaluation of Heilongjiang University of Chinese Medicine (Harbin, Heilongjiang, China) and bred under standard laboratory conditions (temperature, 25 °C; relative humidity, 50%) with free access to chow and water for seven days, afterwards, the rats were fasted for 12 h, but always had free access to water before experiment.

### 3.3. Instruments and UHPLC–MS/MS Conditions

Chromatography separation was conducted on an Agilent 6430 LC system (Agilent, Santa Clara, CA, USA) equipped with a SB-C_18_ RRHD column (150 mm × 2.1 mm, 1.8 μm, Agilent). The mobile phase consisted of acetonitrile–water (90:10, *v*/*v*) containing 5 mM ammonium acetate with an isocratic elution at a rate of 0.4 mL/min. The run time was 3 min for each injection. The column temperature was set at 35 °C. The injection volume was 5 μL.

**Figure 4:**
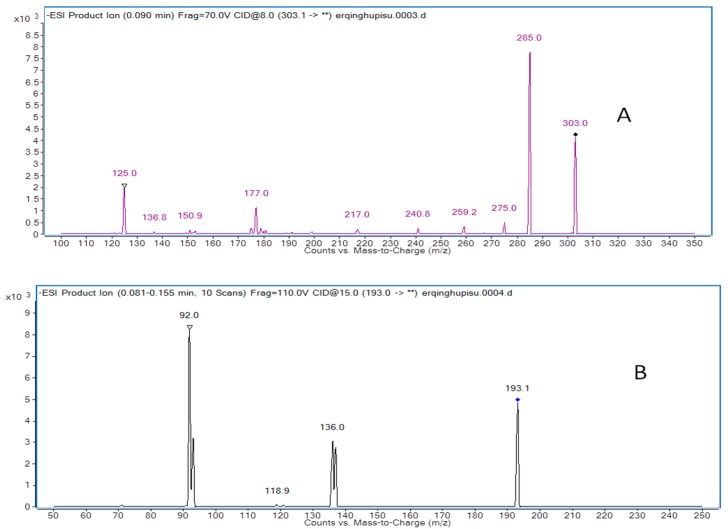
Product ion mass spectra of taxifolin (**A**) and butylparaben (I.S.) (**B**).

Mass spectrometry was operating in negative-ion mode with multiple reaction monitoring (MRM) acquisition [21]. The optimal MS operation parameters are shown in Table 5. High purity nitrogen (N_2_) was used as the nebulizing gas and nitrogen (N_2_) was used as drying gas. The other parameters were exhibited as follows: capillary voltage was set at 4.0 kV, nitrogen was used as desolvation gas at a rate of 500 L/h, and the flow rate of cone gas was 30 L/h. The desolvation gas temperature and source temperature were set at 350 °C and 120 °C, respectively. The precursor-product ion transitions of *m*/*z* at 303.0→285.0 (qualifier of *m*/*z* at 125.0) for taxifolin and 193.1→92.0 (qualifier of *m*/*z* at 136.0) for butylparaben were used for the quantification analysis of taxifolin. Full scans for taxifolin and butylparaben are exhibited in Figure 4.

### 3.4. Preparation of Physical Mixture and Nanodispersions of Taxifolin

The physical mixture of taxifolin was made according to the following preparation method. Taxifolin was mixed at an appropriate amount of compositions (taxifolin:PVP = 1:10). The solids were pulverized, passed through a 200 μm sieve, and stored in a desiccator over a silica gel at room temperature (21 ± 2 °C) before analysis.

The nanodispersion of taxifolin was manufactured strictly according to the literature [14]. Briefly, taxifolin-PVP nanodispersion with a compositions (1:10) was prepared using co-precipitation technique. First, taxifolin was dissolved in water (one part of solid in 10 parts of solvent) in a glass vessel and heated in a water bath with mild stirring. Then, the taxifolin solution was mixed with an appropriate amount of a 20% (*w*/*v*) PVP solution under stirring, cooled and lyophilized. Finally, the solids were pulverized, passed through a 200 μm sieve, and stored in a desiccator over silica gel at room temperature (21 ± 2 °C) before analysis.

### 3.5. Preparation of Standard Solutions and Quality Control Samples

An appropriate amount of taxifolin and butylparaben was dissolved respectively in methanol to make stock solutions at the concentration of 5.0 mg/mL and 2.0 mg/mL. The stock solutions of the standards were further diluted in methanol to produce combined standard working solutions at a series of concentrations. The stock solution of I.S. also diluted with methanol to make the final concentration at 1.0 μg/mL. All samples were stored at 4 °C until to use.

Plasma calibration standards were prepared by spiking the appropriate amounts of the working standard solutions (evaporated to dryness under a stream of nitrogen) into 100 μL blank plasma (vortexed for 30 s) to obtain final concentration levels of 5.0, 10.7, 53.5, 214, 856, 2140,and 4280 ng/mL. The blank plasma was collected from blank rats and compiled to get an enough volume. Quality control (QC) samples at the concentrations of 8, 800, 3400 ng/mL were made by the same way for the validation of the method.

### 3.6. Sample Treatment

Plasma 100 μL (calibration standards, QCs, and unknown samples) followed by 50 μL of I.S. (1.0 μg/mL) and 20 μL of acetic acid was added into a fresh 10 mL clean glass tube. This sample was vortexed thoroughly for 40 s to mix well, then extracted with 3 mL of ethyl acetate by vortex mixing for 1 min and centrifuged at 4000 rpm for 10 min. The upper organic phase was carefully transferred to another clean tube and dried by nitrogen gas at room temperature. The residues were dissolved in 100 μL methanol followed by vortex-mixing and a 5 μL solution was injected into the UHPLC-MS/MS system (Agilent) for analysis [22].

### 3.7. Method Validation

The test method for the determination of taxifolin in rat plasma was validated by following the FDA guidelines (FDA 2001). The method was validated for selectivity, sensitivity, linearity, intra and inter-day precision and accuracy, stability, matrix effects, and extraction recovery. The validation runs were conducted on three consecutive days. Each validation run consisted of two sets of calibration standards and six replicates of QC samples at three concentrations [23,24].

#### 3.7.1. Specificity and selectivity

The specificity of this analytical method was performed by comparing the difference between blank plasma and blank plasma spiked with taxifolin and I.S. The taxifolin standard used was at the lowest concentration on the calibration curve (LLOQ) level so as to be sure there is no endogenous interference observed at the retention times of taxifolin (0.9 min) and I.S. (1.1 min).

#### 3.7.2. Linearity and Range

The calibration curve was counted by fitting peak area ratios of taxifolin to I.S. to corresponding concentrations by a linear equation with a weighting factor of the reciprocal of the concentration (1/x^2^) on three separate days. The concentrations contained seven points (5.0, 10.7, 53.5, 214, 856, 2140, and 4280 ng/mL). The linearity of each calibration curve was determined by plotting the peak area ratio (y) of analytes to I.S. *versus* the nominal concentration (x) of analytes with a weighted (1/x^2^) least square linear regression. The LLOQ was defined as the lowest concentration of the calibration curve with signal-to-noise ≥10 at which the measured precision expressed as relative standard deviation (R.S.D.) was within ±20% and the accuracy expressed as relative error (R.E.) was within ±20%, evaluated by analyzing samples in six replicates.

#### 3.7.3. Precision and Accuracy

A total of 18 QC samples at three concentration levels were selected to evaluate precision and accuracy of this method. Precision was expressed by the intra- and inter-day relative standard deviation (R.S.D) required to be less than 15% and accuracy was determined as the percentage difference between the measured concentration and the true concentration (R.E.) with an acceptance criterion of ±15% for all QC samples.

#### 3.7.4. Matrix Effect and Recovery

The matrix effect of taxifolin was evaluated by comparing the analyte peak areas obtained from plasma samples spiked after extraction to those from the neat standard solutions at the same concentration. Extraction recovery of taxifolin was evaluated at three QC levels and for the I.S. at one concentration by comparing the analyte peak areas obtained from the QC samples with those obtained from blank plasma samples with the analytes spiked into the post-extraction supernatant.

#### 3.7.5. Stability

The stability was assessed by QC samples at three concentration levels (8,800, 3400 ng/mL) with six samples for each concentration under four different conditions which were exhibited as follows: the first condition was short-term stability, in which exposed analytes at room temperature for 12 h; the second was long-term stability, in which the analytes were stored at −20 °C for one month; the third was freeze–thaw stability, in which the analytes were frozen at −20 °C and thawed at 25 °C through three cycles. Finally, the last was post-preparative stability, in which the analytes were stored in the auto sampler at 20 °C for 24 h. All of the stability testing QC samples were determined by using the calibration curve of freshly prepared standard samples.

### 3.8. Pharmacokinetics Study

Eighteen rats were divided into three groups at random and fasted 12 h before experiment but the animals had free access to water during the experiment. The rats in group one were given intravenous administration of taxifolin at 15 mg/kg. The rats in group two were given oral administration of physical mixture of taxifolin at the dose of 15 mg/kg and the rats in last group were given oral administration of the nanodispersion of taxifolin which were equal to taxifolin at 15 mg/kg. All of the solutions, which were employed to oral administration, were dissolved in pure water and the intravenous administration solutions were dissolved in isotonic sodium chloride solution. The blood (200 μL) samples were collected from suborbital vein into heparinized tubes at 0.02, 0.05, 0.08, 0.17, 0.25, 0.33, 0.50, 0.75, 1, 2, 3, and 6 h after dosing and centrifuged at 12,000 rpm for 10 min at 4 °C immediately. Plasma samples were transferred into 1.5 mL clean centrifuge tubes as soon as possible and stored at −20 °C until analysis. The maximum plasma concentration (*C*_max_) and the time of maximum plasma concentration (*T*_max_) were observed directly from the measured data. The elimination rate constant (*K_e_*) was calculated by linear regression of the terminal points in a semi-log plot of the plasma concentration against time. The elimination half-life (*t*_1/2_) was calculated using the formula *t*_1/2_ = 0.693/*K_e_*. The area under plasma concentration-time curve (*AUC*_0-t_) to the last measurable plasma concentration (*C_t_*) was estimated by using the linear trapezoidal rule. The area under the plasma concentration-time curve to time infinity (*AUC*_0-∞_) was calculated as *AUC_0-∞_* = *AUC*_*0*-t_ + *C_t_*/*K_e_* [25].

## 4. Conclusions

A reliable, sensitive, and specific UHPLC-MS/MS method has been developed and validated for the quantification of taxifolin in rat plasma, which used liquid–liquid extraction as a sample preparation procedure. This validated method was precise, robust and accurate with a short analysis time. The nanodispersion of taxifolin offered a new approach which could improve the oral bioavailability of taxifolin significantly, increased the concentration of analyte in rat plasma and extended the time to the peak concentration comparing with administration of physical mixture of taxifolin.

## Figures and Tables

**Table 1:** Extraction recovery and matrix effect of taxifolin and butylparaben (I.S.).

Analytes	Spiked Concentration, ng/mL	Extraction Recovery		Matrix Effects, %	
		Mean ± SD, %	RSD, %	Mean ± SD, %	RSD, %
taxifolin	8	78.5 ± 8.6	10.9	98.2 ± 5.0	5.1
	800	83.5 ± 4.6	5.5	95.2 ± 1.9	2.0
	3400	80.3 ± 6.5	8.1	93.8 ± 3.3	3.5
I.S.	1000	85.8 ± 3.8	4.4	89.5 ± 3.3	3.7

**Table 2:** Intra and inter-day accuracy and precision of taxifolin in rat plasma (*n* = 6).

Spiked Concentration, ng/mL	Intra-Day			Inter-Day		
	Accuracy, %	Measured Concentration, ng/mL, Mean ± SD	Precision, %	Accuracy, %	Measured Concentration, ng/mL, Mean ± SD	Precision, %
8	−2.5	7.8 ± 0.58	7.4	2.5	8.2 ± 0.74	9.0
800	2.9	823.0 ± 34.9	4.2	−5.6	821.2 ± 50.9	6.2
3400	3.8	3530.7 ± 118.5	3.3	2.7	3486.7 ± 129.0	3.7

**Table 3:** Stability summary of taxifolin under different conditions in rat plasma.

Analytes	Spiked Concentration, ng/mL	Stability, % RE			
		Short-Term Stability	Freeze-Thaw Stability	Long-Term Stability	Post-Preparative Stability
taxifolin	8	2.8	6.6	6.5	5.9
	800	1.5	4.8	8.7	3.3
	3400	1.9	5.1	7.0	5.1

**Table 4:** The main pharmacokinetic parameters of taxifolin after intravenous administration and oral administration of taxifolin and its nanodispersion at a dose of 15 mg/kg.

Routes of Administration	Doses, mg/kg	*C*_max_, ng/mL	AUC_0-t_, ng h/mL	AUC_0–∞_, ng h/mL	*T*_max_, h	*T*_1/2_, h
Intravenousadministration	15	38,711.95 ± 4407.15	12,048.59 ± 2157.20	14,829.02 ± 2675.61	-	2.24 ± 0.42
Oral administration of Physical mixture	15	94.79 ± 15.72	59.11 ± 8.62	153.29 ± 13.17	0.08	6.03 ± 1.42
Oral administration of nanodispersion	15	93.92 ± 10.67	90.89 ± 11.76	180.02 ± 46.52	0.17	4.83 ± 2.54

**Table 5:** Optimized mass spectrometric parameters for the LC/MS analysis of taxifolin and butylparaben in MRM mode.

Analyte	Precursor Ion, *m*/*z*	Product Ion, *m*/*z*	Qualifier Ion, *m*/*z*	Fragment, V	Collision Energy, V	Cell Accelerator Voltage, V	Polarity
Taxifolin	303.0	285.0	125.0	70	10	3	Negative
Butylparaben	193.1	92.0	136.0	110	20	7	Negative
